# STAT3 but Not HIF-1α Is Important in Mediating Hypoxia-Induced Chemoresistance in MDA-MB-231, a Triple Negative Breast Cancer Cell Line

**DOI:** 10.3390/cancers9100137

**Published:** 2017-10-14

**Authors:** Hoda Soleymani Abyaneh, Nidhi Gupta, Aneta Radziwon-Balicka, Paul Jurasz, John Seubert, Raymond Lai, Afsaneh Lavasanifar

**Affiliations:** 1 Faculty of Pharmacy and Pharmaceutical Sciences, University of Alberta, Edmonton, AB T6G 2E1, Canada; hoda1@ualberta.ca (H.S.A.); aneta.radziwon@gmail.com (A.R.-B.); jurasz@ualberta.ca (P.J.); jseubert@ualberta.ca (J.S.); 2 Department of Laboratory Medicine and Pathology, University of Alberta, Edmonton, AB T6G 2E1, Canada; nidhi2@ualberta.ca; 3 Department of Oncology, University of Alberta, Edmonton, AB T6G 2E1, Canada; 4 DynaLIFEDx Medical Laboratories, Edmonton, AB T5J 5E2, Canada; 5 Department of Chemical & Materials Engineering, Faculty of Engineering, University of Alberta, Edmonton, AB T6G 1H9, Canada

**Keywords:** hypoxia-induced chemoresistance, HIF-1α, STAT3, cisplatin, cancer stemness

## Abstract

Hypoxia-induced chemoresistance (HICR) is a well-recognized phenomenon, and in many experimental models, hypoxia inducible factor-1α (HIF-1α) is believed to be a key player. We aimed to better understand the mechanism underlying HICR in a triple negative breast cancer cell line, MDA-MB-231, with a focus on the role of HIF-1α. In this context, the effect of hypoxia on the sensitivity of MDA-MB-231 cells to cisplatin and their stem-like features was evaluated and the role of HIF-1α in both phenomena was assessed. Our results showed that hypoxia significantly increased MDA-MB-231 resistance to cisplatin. Correlating with this, intracellular uptake of cisplatin was significantly reduced under hypoxia. Furthermore, the stem-like features of MDA-MB-231 cells increased as evidenced by the significant increases in the expression of ATP-binding cassette (ABC) drug transporters, the proportion of CD44^+^/CD24^−^ cells, clonogenic survival and cisplatin chemoresistance. Under hypoxia, both the protein level and DNA binding of HIF-1α was dramatically increased. Surprisingly, siRNA knockdown of HIF-1α did not result in an appreciable change to HICR. Instead, signal transducer and activator of transcription 3 (STAT3) activation was found to be important. STAT3 activation may confer HICR by upregulating ABC transporters, particularly ABCC2 and ABCC6. This study has demonstrated that, in MDA-MB-231 cells, STAT3 rather than HIF-1α is important in mediating HICR to cisplatin.

## 1. Introduction

Hypoxia commonly occurs in solid tumors, as a result of their rapid proliferation that outpaces the oxygen supply [1]. In response to hypoxia, cancer cells are known to upregulate a protein called hypoxia inducible factor (HIF) [2]. HIF is a heterodimeric transcription factor comprised of an oxygen regulated unit, HIF-1α, as well as a constitutionally expressed beta unit, HIF-1β. In the presence of oxygen, HIF-1α is degraded by prolyl hydroxylase via ubiquitination and proteolysis [3]. Under hypoxia, HIF-1α is stabilized. After its dimerization with HIF-1β, the HIF heterodimer translocates to the nucleus where it activates the transcription of various downstream targets, many of which are known to be involved in cancer progression, survival, aggressiveness and chemoresistance [2,3,4]. To induce drug resistance, HIF has been demonstrated to reduce drug-induced apoptosis and senescence [5,6] and to induce autophagy [7,8]. In view of its significance as a master regulator of hypoxia-induced chemoresistance (HICR), HIF-1, especially its α subunit, is considered to be a therapeutic target for cancer treatment [5,6,7,9,10,11,12].

While HIF has been shown to be the key mediator of HICR, a relatively small number of publications have implicated other molecular mechanisms in conferring HICR. For instance, p53 can be inactivated in cancer cells in hypoxia, inducing resistance to p53-mediated apoptosis [13,14,15]. It has also been shown that hypoxia can induce the activation of a number of signaling pathways, including those of phosphoinositol-3-kinase (PI3K), nuclear factor kappa-B (NFκB), cycloxygenase-2 (COX-2), activator protein-1 (AP-1), c-Jun, Pim-1, apoptosis inhibitory protein (IAP-2) and signal transducer and activator of transcription 3 (STAT3) protein, and these hypoxia-induced biochemical alterations may contribute to drug resistance under hypoxic conditions [13,16,17,18,19,20,21,22,23,24]. However, in most of these studies, the role of HIF-1α relative to that of these signaling pathways was not directly assessed. Specifically, it is not clear if these pathways are key mediators of HICR that is independent of HIF-1α. 

In this study, we aimed to understand the mechanism underlying HICR in triple negative breast cancer (TNBC), since TNBC tumors are known to frequently carry a hypoxic phenotype and HIF-1α is known to be frequently over-expressed in these tumors [25]. Cisplatin, a platinum used as part of the standard chemotherapy regimen for TNBC patients [25,26], was used in this study. Our findings suggest that STAT3, instead of HIF-1α, is the key player of HICR to cisplatin in MDA-MB-231, a TNBC cell line. This study has demonstrated that, while HIF-1α is recognized to be an important mediator for HICR, exceptions exist.

## 2. Results

### 2.1. Hypoxia Induces Resistance to Cisplatin in MDA-MB-231 Cells

**Figure 1:**
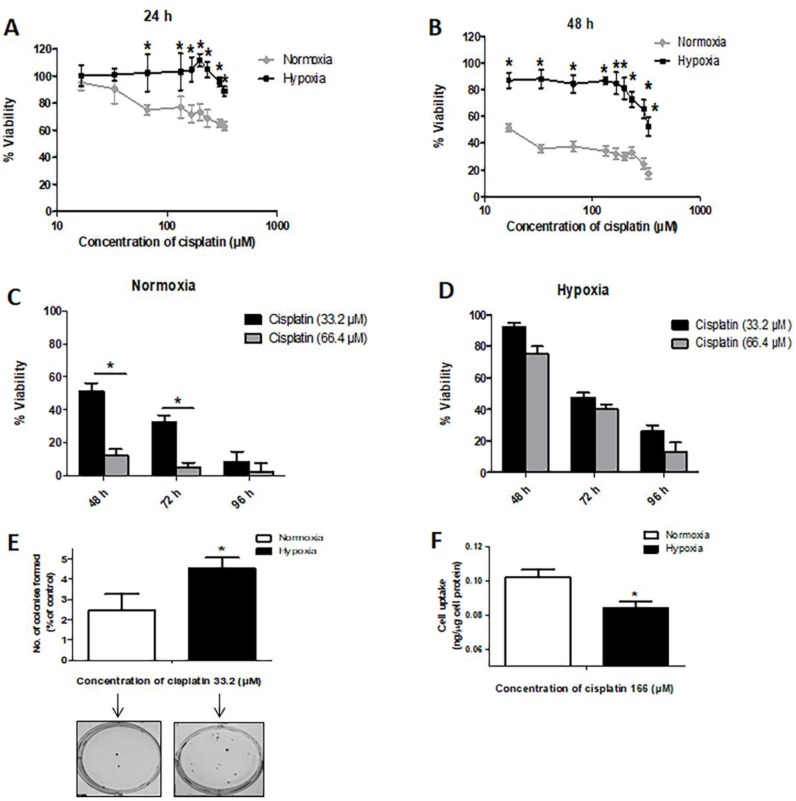
Hypoxia confers chemoresistance to cisplatin in MDA-MB-231 cells. Drug sensitivity of cells under normoxia or hypoxia was measured by MTT (3-(4,5-dimethylthiazol-2-yl)-2,5-diphenyltetrazolim bromide assay) for (**A**) 24 h and (**B**) 48 h treatment with cisplatin. (*) denotes a significant difference compared to the normoxic group at each individual cisplatin concentration (Student’s *t* test, *p* < 0.05). The viability of MDA-MB-231 cells was measured by trypan blue assay for cells treated with cisplatin (33.2 and 66.4 µM) for 48, 72 and 96 h under (**C**) normoxia and (**D**) hypoxia. (*) denotes a significant difference compared to lower cisplatin concentrations (33.2 µM) at each time point (Student’s *t* test, *p* < 0.05). (**E**) Clonogenic survival assay was conducted for cells treated with cisplatin (33.2 µM) under hypoxia or normoxia (24 h) in duplicate with plating of 500 cells. The number of colonies formed (% of control) from 500 cells was graphed. (**F**) Cisplatin cellular uptake was measured by an ion coupled plasma mass spectrometer (ICP-MS) for 24 h treatment with cisplatin. (*) denotes a significant difference between normoxic and hypoxic groups (Student’s *t* test, *p* < 0.05). Data are represented as mean ± SD (*n* = 3).

Using the MTT (3-(4,5-dimethylthiazol-2-yl)-2,5-diphenyltetrazolim bromide) assay to quantify the number of viable cells, we found that hypoxia significantly increased the resistance of MDA-MB-231 cells to cisplatin (Figure 1A,B), increasing the inhibitory concentration at 50% (IC_50_) from 17 µM (as seen under normal conditions) to 330 μM at 48 h after cisplatin treatment (Figure 1B). Similar results were obtained when trypan blue assay was used to assess the number of viable cells (Figure 1C,D). As shown in Figure 1E, we performed clonogenic survival assays; with cisplatin treatment, we found that cells cultured under hypoxia had a significantly higher number of colonies than cells grown under the normal condition. Of note, without cisplatin treatment, there was no significant difference in the clonogenic potential between cells grown in hypoxia or normal conditions (Supplementary Materials Figure S1). Correlating with our observation that hypoxia induces chemoresistance in MDA-MB-231 cells, we found that the cell uptake of cisplatin under hypoxia was significantly less than that under the normal condition (Figure 1F). The intracellular cisplatin level was measured by using an ion-coupled plasma mass spectrometer, as detailed in Materials and Methods.

### 2.2. Hypoxia Confers Stem-Like Features to Cells

**Figure 2:**
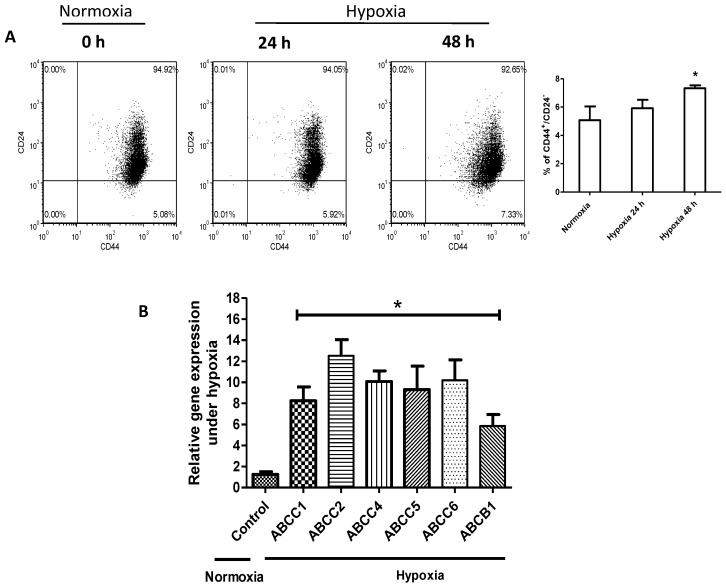
Hypoxia confers stem-like features to cells. The effect of hypoxia in the MDA-MB-231 cells on (**A**) the enrichment of cells with CD44^+^/CD24^−^ subpopulation after 24 and 48 h incubation under hypoxia. (*) denotes a significant difference as compared to the normoxic group (Student’s *t* test, *p* < 0.05) and (**B**) qRT-PCR results of ATP-binding cassette (ABC) drug transporters gene expression after 48 h hypoxia normalized to glyceraldehyde 3-phosphate dehydrogenase (GAPDH), and further normalized to the normoxic group. (*) denotes a significant difference as compared to the normoxic group (Student’s *t* test, *p* < 0.05). Data are represented as mean ± SD (*n* = 3).

It has been previously published that hypoxic challenge can enrich the cancer stem cell (CSC) population in MDA-MB-231 [27]. As shown in Figure 2A, we identified a significant increase in the proportion of cells expressing the CD44^+^/CD24^−^ immunophenotype, a well-documented CSC marker [28]. In further support of the concept that hypoxia promotes cancer stemness, we found that hypoxic challenge resulted in a significant increase in the mRNA levels of ATP-binding cassette (ABC) drug transporters (i.e., ABCC1–6 as well as ABCB1) (Figure 2B), previously shown to be CSC markers [29].

### 2.3. HIF-1α Is Upregulated and Functionally Active in Response to Hypoxia

**Figure 3:**
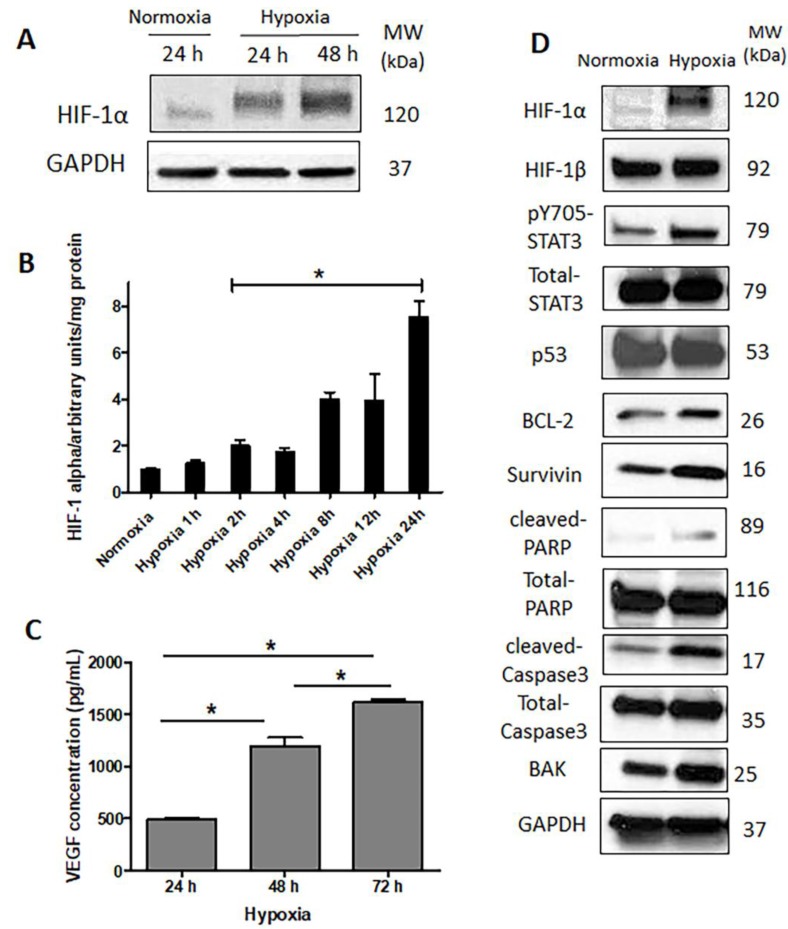
Hypoxia inducible factor-1α (HIF-1α) is upregulated and functionally active in response to hypoxia. The effect of hypoxia on (**A**) the expression of HIF-1α protein for different time periods, and (**B**) activation (DNA binding) of HIF1-α. (*) denotes a significant difference compared to the normoxic group (Student’s *t* test, *p* < 0.05). (**C**) vascular endothelial growth factor (VEGF) production. (*) denotes groups are significantly different from each other (one way ANOVA (analysis of variance) followed by a post-hoc Tukey test, *p* < 0.05). (**D**) Expression of HIF-1α related proteins after 48 h incubation under hypoxia and normoxia. Data are represented as mean ± SD (*n* = 3). Representative results of three independent Western blot analyses are shown.

As expected, hypoxic challenge led to a substantial increase in the expression of HIF-1α protein (Figure 3A). Moreover, the functional status of HIF-1α increased, as the DNA-binding of this protein showed a significant and time-dependent increase, detectable as early as 2 h after the initiation of the hypoxic challenge (Figure 3B). In accordance with these observations, vascular endothelial growth factor (VEGF), a known downstream target of HIF-1α, also showed a time-dependent increase in its secreted levels at 72 h under hypoxia (Figure 3C). As shown in Figure 3D, hypoxia also upregulated the expression of several downstream targets of HIF-1α that have known anti-apoptotic functions, such as survivin and BCL-2 (B-cell lymphoma 2). While we identified increases in the expression in BAK (BCL-2 homologous antagonist/killer), cleaved caspase3 and cleaved PARP (poly (ADP-ribose) polymerase), these changes were relatively subtle, suggesting a low degree of apoptosis induced by hypoxia (Figure 3D).

Since STAT3 has been shown to stabilize HIF-1α upon hypoxia in Caki-1, a human renal carcinoma cell line [30] and MCF-7, an estrogen receptor-positive breast cancer cell line [31], we asked if STAT3 is activated in our experimental model. As shown in Figure 3D, hypoxic challenge induced a substantial upregulation of phospho-STAT3 (pSTAT3), the active form of STAT3. Of note, in contrast to HIF-1α, HIF-1β did not change in response to hypoxia. This finding is in keeping with the concept that it is the constitutively expressed subunit of HIF.

### 2.4. Hypoxia-Induced STAT3 Activation Is Independent of HIF-1α

**Figure 4:**
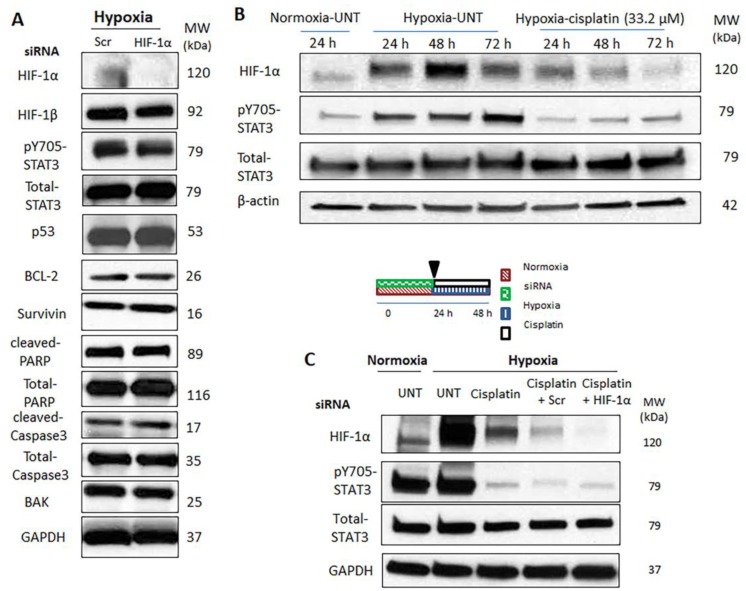
Hypoxia-induced STAT3 (signal transducer and activator of transcription 3) activation is independent of HIF-1α (hypoxia inducible factor-1α). Expression of HIF-1α related proteins was measured following (**A**) HIF-1α/scrambled (Scr) siRNA treatment under hypoxia. Cells were transfected with HIF-1α/Scr siRNAs under normoxia (24 h) and then kept under hypoxia (48 h), (**B**) cisplatin treatment (33.2 µM) under hypoxia (24–72 h), and (**C**) HIF-1α/Scr siRNAs and cisplatin treatment under hypoxia. Cells were transfected with HIF-1α/Scr siRNAs under normoxia (24 h) and then treated with cisplatin (33.2 µM) under hypoxia (24 h). Representative results of three independent Western blot analyses are shown. Densitometry analysis is shown in Supplementary Materials Figure S2.

To assess if HIF-1α plays a direct role in the observed biological changes induced by hypoxia, we knocked down the expression of HIF-1α using siRNA. As shown in Figure 4A, successful knockdown of HIF-1α was achieved. However, we did not observe appreciable changes in the levels of survivin, BCL-2, and BAK, all of which are downstream targets of HIF-1α. Accordingly, no detectable changes in the levels of cleaved caspase3 and cleaved PARP expression were identified. Importantly, pSTAT3 was still expressed at a relatively high level, indicating that the hypoxia-induced upregulation of pSTAT3 is not dependent on HIF-1α (Figure 4A). 

Since it has been reported that cisplatin can exert an inhibitory effect on HIF-1α [32], we will further address the question of whether STAT3 activation induced by hypoxia is dependent on HIF-1α. Thus, MDA-MB-231 cells were exposed to cisplatin at a relatively low concentration (32 μM) under hypoxia for different time durations (i.e., 0–72 h). As shown in Figure 4B, in the absence of cisplatin (i.e., without HIF-1α inhibition), we observed upregulations of HIF-1α and pSTAT3 upon hypoxic challenge. In comparison, with cisplatin treatment (i.e., with HIF-1α inhibition), the hypoxia-induced upregulation of pSTAT3 was sustained, despite the suppression of HIF-1α by cisplatin. The combination of both HIF-1α siRNA knockdown and cisplatin treatment revealed similar results under hypoxia (Figure 4C). Taken together, it appears that hypoxia-induced STAT3 activation is not dependent on HIF-1α.

### 2.5. Simultaneous Inhibition of HIF-1α and STAT3 Proteins Is More Efficient in Suppressing the Acquisition of Cancer Stemness Induced by Hypoxia

**Figure 5:**
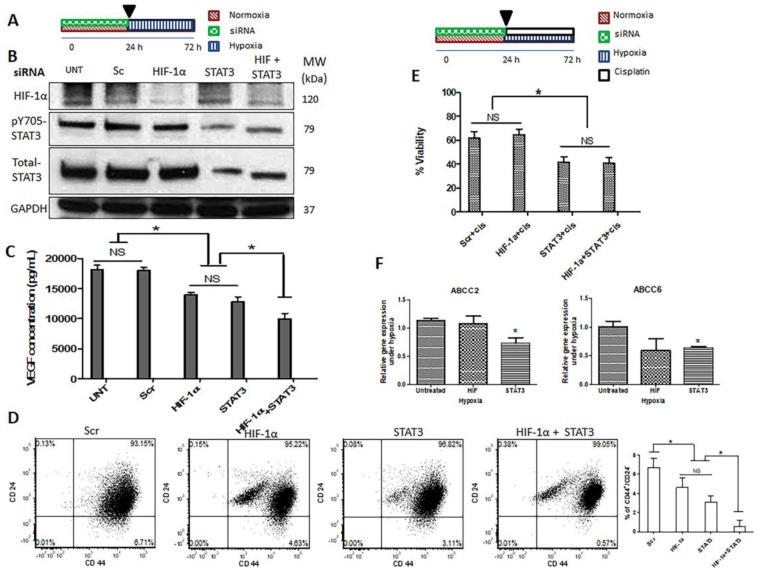
Simultaneous inhibition of HIF-1α (hypoxia inducible factor-1α) and STAT3 (signal transducer and activator of transcription 3) proteins is more efficient in suppressing the acquisition of cancer stemness induced by hypoxia. (**A**) siRNA treatment diagram for (**B**–**D**). MDA-MB-231 cells were transfected with HIF-1α and STAT3 siRNAs individually or combined under normoxia (24 h) and then incubated under hypoxia (48 h), (**B**) Successful knockdown of HIF-1α and STAT3 as shown by Western blot analyses, (**C**) vascular endothelial growth factor (VEGF) production, (**D**) Expression of CD44^+^/CD24^−^ were measured for cells with flow cytometry. (**E**) Viability of cells was measured by MTT assay after cells were transfected with HIF-1α and STAT3 siRNA individually or combined under normoxia (24 h) and then treated with cisplatin (66.4 µM) under hypoxia (48 h). (*) denotes a significant difference between the groups (one way ANOVA (analysis of variance) followed by a post-hoc Tukey test, *p* < 0.05). (**F**) qRT-PCR results of ABCC2 and ABCC6 expression in the MDA-MB-231 cells. Cells were treated with scrambled (Scr), HIF-1α, and STAT3 siRNAs under normoxia (24 h), and then incubated under hypoxia (48 h). The qRT-PCR results were normalized to glyceraldehyde 3-phosphate dehydrogenase (GAPDH), and further normalized to the untreated hypoxic sample. (*) denotes a significant difference as compared to the untreated hypoxic group (Student’s *t* test, *p* < 0.05). Data are represented as mean ± SD (*n* = 3).

In view of the fact that HIF-1α and STAT3 are known to promote cell viability in face of adversity, our collected data has led us to propose a model in which these two molecules work as two redundant systems in maintaining cell viability and tumorigenicity under hypoxia. To provide evidence to support this concept, we knocked down HIF-1α and STAT3 simultaneously (Figure 5B). If our model is correct, one would expect to observe that the simultaneous inhibition of these two proteins is more efficient in suppressing the acquisition of cancer stemness induced by hypoxia, as compared to inhibition with either protein alone. As shown in Figure 5C, simultaneous inhibition of both proteins resulted in a significant downregulation of VEGF expression, as compared to the inhibition of either protein alone. Moreover, we found that the simultaneous knockdown of both HIF-1α and STAT3 resulted in a significant decrease in the proportion of cells expressing the CD44^+^/CD24^−^ immunophenotype, as compared to the inhibition of either protein alone (Figure 5D).

We also performed an MTT assay to compare single siRNA knockdown of either HIF-1α/STAT3 to the simultaneous knockdown of both proteins. As shown in Figure 5E, we found that the double knockdown was efficient in decreasing the number of viable cells after cisplatin treatment, although the decrease was not substantially different from that of the single knockdown of STAT3. Of note, siRNA knockdown of HIF-1α under hypoxia did not substantially reverse the HICR to cisplatin (Supplementary Materials Figures S3–S5). In addition, stabilization of HIF-1α in normoxia using cobalt chloride did not induce resistance to cisplatin (Supplementary Materials Figure S6).

We postulated that the HICR to cisplatin in MDA-MB-231 cells may be due to an upregulation of ATP-binding cassette (ABC) drug transporters (i.e., ABCC1, ABCC2, ABCC5 and ABCC6), as these molecules have been implicated in cisplatin resistance in other cell types [33,34]. Thus, we investigated the effect of siRNA knockdown of HIF-1α or STAT3. As shown in Figure 5F, only the knockdown of STAT3 resulted in a significant reduction in the mRNA expression levels of ABCC2 and ABCC6 under hypoxia; in contrast, the knockdown of HIF-1α did not appreciably change the expression levels of these transporters. As for ABCC1 and ABCC5, we observed a decreased level of gene expression after STAT3 knockdown, although the levels of reduction were not statistically significant (Supplementary Materials, Figure S7).

## 3. Discussion

Hypoxia-induced chemoresistance (HICR) has been observed in a number of human cancer models [22,35,36] including TNBC [37,38,39]. While the mechanism is not well understood, the role of HIF-1α has been highlighted [5,6,7,9,10,11,38,40,41,42,43,44,45]. To induce chemoresistance under hypoxia, it has been shown that HIF reduces drug-induced apoptosis and senescence [5,6] and induces autophagy [7,8]. In this study, we aimed to expand on our understanding of HICR. We employed an experimental model in which a TNBC cell line, MDA-MB-231, was treated with cisplatin, which is a front-line chemotherapeutic agent used to treat TNBC patients [25,26]. Consistent with the published results of several studies using a variety of cancer cell lines and cisplatin under hypoxic conditions [21,35,46,47,48], we found that hypoxia significantly induced cisplatin resistance in MDA-MB-231 cells. Importantly, these findings correlated with a reduced cellular uptake of cisplatin and increased stem-like features in these cells, as evidenced by the significant increase in the expression level of ABC transporters and the significantly higher proportions of cells expressing CD44^+^/CD24^−^. To our knowledge, this hypoxia-induced increase in stem-like features have been described in previously published studies of human cancer cells [49,50,51]. Overall, we believe that our experimental model is valid and appropriate to study HICR in cancer cells. 

HIF-1α is considered to be a major factor in HICR [5,6,7,9,10,11,38,40,41,42,43,44,45]. In a wide range of different tumor types, HICR was shown to be reversed by HIF-1α inhibition [5,6,9,10,38,41,42,43,45]. Our findings regarding the role of HIF-1α in HICR to cisplatin in MDA-MB-231 cells is however rather unexpected. Specifically, while we found that HIF-1α was effectively upregulated by hypoxia, siRNA knockdown of this protein did not significantly modulate the level of chemoresistance to cisplatin, nor the expression levels of a panel of apoptotic proteins, suggesting the involvement of other mechanisms that are independent of HIF-1α in the context of hypoxia-induced cisplatin resistance. In concert with the published data, upregulation of HIF-1α is not sufficient to increase chemoresistance [15,22], since stabilization of HIF-1α in normal conditions using cobalt chloride did not induce cisplatin chemoresistance.

HIF-1α independent mechanisms of drug resistance in hypoxia are rarely reported. We have found similar observations published in only four studies [15,18,22,52]. In all of these studies, inhibition of HIF-1α either using siRNA, short hairpin RNA interference (shRNAi) or a dominant negative construct did not lead to a significant reduction in HICR. Instead of HIF-1α, a number of other mechanisms were highlighted as potential ‘substitutes’ for HIF-1α in conferring chemoresistance, including AP-1 induction, p53 suppression and mitochondrial inhibition [15,18,22,52]. None of these four studies employed TNBC in their experimental models. To our knowledge, the role of HIF-1α in conferring HICR in TNBC has not been previously studied in detail. In view of the fact that most published studies support the importance of HIF-1α in conferring HICR, we considered two possible explanations for this apparent discrepancy. First, we considered that the role of HIF-1α is variable depending on the cell types used in the study. Second, the exact chemotherapeutic agents used in the study are likely to be relevant. For instance, cisplatin is known to have inhibitory effect on HIF-1α function [32].

The high activation level of STAT3 under hypoxia, an oncoprotein strongly implicated in chemoresistance in cancer cells [34,53,54,55], has led us to hypothesize that STAT3 sustained the chemoresistant phenotype despite the effective experimental abrogation of HIF-1α. STAT3 activation has been recognized as one of the mechanisms that confer chemoresistance in hypoxic condition [21]. In another study using ovarian cancer cells treated with cisplatin under hypoxia, the authors also highlighted the role of STAT3 in this context [21], however whether HIF-1α is important was not examined. In support of the importance of STAT3 in this context, we found treatment of cells with STAT3 siRNA to be highly effective in reversing hypoxia-induced chemoresistance to cisplatin, whereas HIF-1α siRNA was relatively ineffective. The importance of STAT3, relative to that of HIF-1α, was highlighted by the observation that the simultaneous inhibition of these two proteins did not result in a significantly higher reversal of chemoresistance compared to STAT3 inhibition alone. Of note, we admit that a potential shortcoming of this study is the inclusion of only one STAT3 siRNA. Nonetheless, this specific STAT3 siRNA species was validated in one of our earlier studies [56]. 

The inter-relationship between the expression of HIF-1α and STAT3 has been previously studied. Relatively extensive evidence has been published in support of the concept that STAT3 regulates the expression/function of HIF-1α [31]. Furthermore, it has been shown that STAT3 works with HIF-1α; for instance, STAT3 is known to be part of the HIF-1α-DNA complex, which mediates the gene transcription functions of HIF-1α [57]. Nonetheless, not much is known as to whether the expression and/or activation of STAT3 is regulated by HIF-1α. In our model, the observation that STAT3 remained highly activated after the siRNA knockdown of HIF-1α strongly suggests that STAT3 activation in response to hypoxia is not dependent on HIF-1α. This is in parallel with the results of a previous study, which has shown the same phenomenon in MDA-MB-231 cells [57]. Nonetheless, this conclusion may be specific to cell types and/or experimental conditions, since pSTAT3 upregulation independent of HIF-1α has been previously reported in a human hepatoma cell line [58] while STAT3 down-regulation secondary to HIF-1α knockdown has also been found in human colon cancer cells [59].

The multi-drug resistance related protein (MRP) family of the ATP-binding cassette (ABC) drug transporters (ABCC1-13), breast cancer resistance protein BCRP (ABCG2) and ABCB1 (MRD1, p-gp) are the best-known transporters mediating the multi-drug resistance phenotype. Whereas it has been clearly established that ABCB1 and ABCG2 do not confer resistance to platinum compounds, selected numbers of the MRP family have been implicated in resistance to cisplatin [33].

Our data has led us to hypothesize that STAT3 activation may confer chemoresistance under hypoxia via its upregulation of a selected number of ABC drug transporters, particularly those members that have been implicated in resistance to cisplatin [33]. STAT3 knockdown resulted in significantly lower level of expression of ABC drug transporters involved in cisplatin resistance (i.e., ABCC2 and ABCC6); however, knockdown of HIF-1α did not change the expression levels of these transporters under hypoxia. These results may explain why STAT3 knockdown is more effective in reversing hypoxia-induced cisplatin resistance as compared to HIF-1α in MDA-MB-231.

Of note, reduced intracellular cisplatin accumulation due to hypoxia-induced upregulation of ABC drug transporters is one of the many mechanisms responsible for the development of cisplatin resistance. For instance, an increased level of the antioxidant glutathione (GSH) is another known hypoxia-induced adaptation for protecting tumor cells against oxidative stress and can confer drug resistance to cisplatin by its inactivation [60].

We would like to point out that we performed most of the described experiments using another TNBC cell line, SUM149, but we found that the results were not entirely consistent with those of MDA-MB-231. Thus, the results described here are either specific to MDA-MB-231 and/or the knowledge generated may be applicable to only a subset of TNBC tumors.

How STAT3 confers HICR also may be related to its ability to upregulate CD44, a marker of cancer stemness [61,62]. Previous studies have shown that STAT3 can function as a modulator for CD44 expression in aggressive breast cancer cells and promote the CSC phenotype [62]. Notably, it has been demonstrated that CD44^+^ breast cancer stem cells had a relatively high level of STAT3 activation [63]. Furthermore, STAT3-mediated tamoxifen resistance also has been shown in the CD44^+^/CD24^−/low^ subpopulation of MCF-7 breast cancer stem cells characterized by their high mammosphere formation capacity [61].

## 4. Materials and Methods

### 4.1. Cell Culture

TNBC cell line, MDA-MB-231 was obtained from ATCC (Manassas, VA, USA) and maintained in Roswell Park Memorial Institute medium (RPMI) 1640 medium supplemented with 10% fetal bovine serum (Invitrogen, Karlsruhe, Germany), 100 units/mL penicillin, and 100 mg/mL streptomycin in a humidified incubator under 95% air and 5% CO_2_ at 37 °C. For the hypoxic condition, cells were cultured in a CO_2_ incubator maintained at 94% N_2_, 5% CO_2_ and 1% O_2_. MDA-MB-231 cells were plated at 25–30% confluence and cultured until they reached 60–70% confluence for different treatments. The chemotherapeutic agent cisplatin (cis-diamminedichloroplatinum(II) (CDDP) (purity 99%), #H878, AK Scientific Inc., Union City, CA, USA) was freshly prepared in water as a stock solution (3.3 mM) and further diluted with the RPMI 1640 medium to reach the indicated concentrations. 

### 4.2. Small Interfering RNAs (siRNAs) Complex Preparation

HIF-1α siRNAs (Hs_HIF1α_5 FlexiTube siRNA, #SI02664053, Qiagen, Hilden, Germany) [64], scrambled (Scr) siRNAs (Negative Control siRNA, #1027310, Qiagen), STAT3 siRNAs (Hs_STAT3_7 FlexiTube siRNA, #SI02662338, Qiagen) [56] and Lipofectamine™ 2000 Transfection Reagent (Invitrogen, Carlsbad, CA, USA), were used to make complexes with a (siRNA:polymer) ratio of 1:1 (weight/weight) in OptiMEM media (Life Technologies, Grand Island, NY, USA) according to the manufacturer’s instructions. Complexes were added to the cells at 50 nM siRNA concentrations. Cells were transfected with siRNA complexes at 50–60% confluence.

### 4.3. Trypan Blue Assay

MDA-MB-231 cells (7 × 10^4^ cells/well) were seeded in 24-well plates (1 mL in each well) overnight, then exposed to cisplatin at the indicated concentrations and incubated under normoxia or hypoxia. Then the floating and adherent cells (harvested by trypsinization) were collected separately at different time points and re-suspended in trypan blue solution (0.4%) (Sigma-Aldrich, Oakville, ON, Canada) and the number of viable and dead cells was counted in a hemocytometer under a light microscope. At least 100 cells were counted for each sample. The percentage of viable cells is presented as the mean ± SD for three independently performed experiments.

### 4.4. MTT Assay

MDA-MB-231 cells (1 × 10^4^ cells/well) were seeded in 96-well plates overnight and then exposed to increasing concentrations of cisplatin (3.32–332 µM) for 24 and 48 h under hypoxia or normoxia. For siRNA transfection studies, cells (7 × 10^4^ cells/well) were seeded in 24-well plates overnight. Then, cells at 50–60% confluence were transfected with siRNA complexes prior to cisplatin treatment for 24 h under normoxia. Cellular viability was assessed by the reduction of MTT (3-(4,5-dimethylthiazol-2-yl)-2,5-diphenyltetrazolim bromide, Sigma-Aldrich, Oakville, ON, Canada) to formazan crystals. Briefly, MTT solution (5 mg/mL) was added to incubated cells for 4 h at 37 °C prior to assessment. Then the medium was replaced by N,N,dimethyl sulfoxide (DMSO) to dissolve the crystals formed. Optical density was measured spectrophotometrically using a plate reader (Synergy H1 Hybrid Reader, Biotek, Winooski, VT, USA) at 570 nm. The cellular activity ratio was represented relative to control.

### 4.5. Colony Formation Assay

MDA-MB-231 cells (35 × 10^4^ cells/flask) were seeded in 25 cm^2^ flasks overnight. The day after, the culture medium was replaced with either drug-free medium (for non-treated controls) or medium containing cisplatin. Drug exposure was performed either under normoxia or hypoxia for 24 h. Cells were then washed once in 1× phosphate buffered saline (PBS), harvested by trypsinization, counted using a hemocytometer, and then re-plated at three different densities of 500, 1000, and 2000 cells/well in duplicate in six-well plates under normoxia. After an additional seven to 10 days of culture, cells were stained with a crystal violet solution (#HT90132, Sigma-Aldrich, St. Louis, MO, USA), and surviving colonies consisting of ~50 or more cells were counted with a Protein Simple, Alpha Imager HP. In another set of experiments, cells were first transfected with siRNA complexes for 24 h under normoxia and then treated with cisplatin under hypoxia for another 24 h and then the above procedure was repeated.

### 4.6. Flow Cytometry Analyses for CD44^+^/CD24^−^ Expression

Single cell suspensions for flow cytometry were achieved by passing the cells through a 40 μm cell strainer (BD Falcon, BD Biosciences, Franklin Lakes, NJ, USA) and staining with CD44-APC (#559942) and CD24-PerCP-Cy5.5 (#561647) from BD Pharmingen in Hanks’ buffer supplemented with 2% fetal bovine serum (FBS) according to the manufacturer’s instructions. All stained cells were run in a BD FACS Canto II (BD Biosciences, San Jose, CA, USA), and data were analyzed using FCS Express 5.0 software (De Novo Software, Glendale, CA, USA). To assess the changes in the expression of CD44^+^/CD24^−^ under hypoxia, as compared to normoxia, gates were first established for positivity stained normoxic cells with antibodies of CD44 and CD24 using unstained normoxic cells as a negative control. Then, the stained normoxic group was chosen as a control and the same gating was adopted to measure the expression of CD44^+^/CD24^−^ in hypoxic cells or other treatments.

### 4.7. RNA Extraction, cDNA Synthesis, Quantitative Reverse Transcription Polymerase Chain Reaction (qRT-PCR)

Total RNA extraction was performed with the Qiagen RNeasy Kit (#74104 Qiagen) according to the manufacturer’s protocol. A total of 1 µg of RNA was reverse transcribed using oligo-dT and superscript II (Life Technologies, Grand Island, NY, USA) according to the manufacturer's protocol. Then 1 µL of the resulting cDNA mixture was added to the Platinum SYBR Green qPCR SuperMix-UDG with Rox (Life Technologies, Grand Island, NY, USA) and amplified with target gene-specific primers (as shown in Table 1) on the Applied Biosystems 7900HT (Carsbad, CA, USA; The Applied Genomics Centre, Edmonton, AB, Canada). All genes of interest were normalized to glyceraldehyde-3-phosphate dehydrogenase (GAPDH) transcript expression levels. For analysis of changes in gene expression under hypoxia, fold changes in gene expression were calculated using the 2^−ΔΔCT^ method. Individual fold-changes for each of the hypoxic samples were calculated by subtracting the ΔCT (gene expression cycle threshold (CT) normalized to the endogenous control, GAPDH) for each sample from the average ΔCT for the normoxic group to obtain ΔΔCT and was entered into the formula 2^−ΔΔCT^ to obtain the fold changes.

### 4.8. Western Blot

To measure the expression level of different proteins, MDA-MB-231 cells (20 × 10^4^ cells/well) were seeded in six-well plates overnight. Then cells were transfected with siRNAs for 24 h under normoxia. Then after 48 h incubation under hypoxia, cells were washed with cold 1× PBS and lysed using radioimmunoprecipitation assay buffer (RIPA) lysis buffer that was supplemented with 0.1 mM phenylmethylsulfonyl fluoride (PMSF) (Sigma-Aldrich), a protease Inhibitor Cocktail Set III, Animal-Free—Calbiochem (#535140, Millipore, Billerica, MA, USA), and a phosphatase Inhibitor Cocktail Set II (#524625, Millipore). The lysate was then incubated on ice for 30 min, which was followed by centrifugation at 17,000 *g* for 20 min to remove genomic DNA. Protein quantification was determined by the bicinchoninic acid (BCA) protein assay kit (Pierce, Rockford, IL, USA), and equal amounts of protein (35–40 μg) were loaded in 4–15% Tris-Glycine gradient gel (#456-1084, Biorad, Pleasanton, CA, USA). After gel electrophoresis, proteins were transferred to a nitrocellulose membrane. Membranes were probed with antibodies against HIF-1α (#3716s, Cell Signaling Technologies, Danvers, MA, USA), Hypoxia Inducing Factor-1β (HIF-1β) (#sc-8076, Santa Cruz Biotechnologies, Dallas, TX, USA), survivin (#2808s, Cell Signaling Technologies), and B-cell lymphoma 2 (BCL-2) (#sc-130308, Santa Cruz Biotechnologies), cleaved poly (ADP-ribose) polymerase (c-PARP) (#9544s, Cell Signaling Technologies), PARP (#9542, Cell Signaling Technologies), phospho-STAT3 (Tyr705) (p-STAT3) (#9131, Cell Signaling Technologies), Total-STAT3 (T-STAT3) (#8768s, Cell Signaling Technologies), c-Myc (#5605s, Cell Signaling Technologies), cleaved caspase-3 (#9661s, Cell Signaling Technologies), caspase-3 (#9662s, Cell Signaling Technologies), BCL-2 homologous antagonist/killer (BAK) (#3814s, Cell Signaling Technologies), p53 (#554293, BD Pharmingen, BD Biosciences), GAPDH (# sc-47724, Santa Cruz Biotechnologies) and β-actin (#sc-47778, Santa Cruz Biotechnology). Proteins were then detected using peroxidase-conjugated anti-mouse IgG (#7076, Cell Signaling Technologies) or anti-rabbit IgG (#7074, Cell Signaling Technologies) and visualized by enhanced chemiluminescence (Pierce ECL Western Blotting Substrate, #32106, Thermo Scientific, Rockford, IL, USA).

### 4.9. Vascular Endothelial Growth Factor Enzyme-Linked Immunosorbent (VEGF Elisa) Assay

The level of secreted VEGF was determined by the Quantikine Human VEGF Immunoassay kit (#SVE00, R&D Systems, Minneapolis, MN, USA). Briefly, cells were left untreated or transfected by siRNA under normoxia for 24 h and then kept under hypoxia for additional 48 h. The supernatant was then removed and analyzed for VEGF levels in pg/mL according to the manufacturer’s instructions.

### 4.10. HIF-1α DNA Binding Activity

HIF-1α DNA binding activity was measured in a nuclear extract by HIF-1α transcription factor assay Abcam Kit (#ab133104, Abcam, Cambridge, UK). Briefly, cell lysates for samples incubated under normoxia or hypoxia for different incubation times were collected. The HIF transcription factor complex present in the nuclear extract was then detected according to the manufacturer’s instructions.

### 4.11. Flow Cytometric Detection of Apoptosis Using Annexin V-FITC and Propidium Iodide

Annexin V-FITC (Fluorescein IsoThioCyanate) and propidium iodide (PI) from BD Biosciences (FITC Annexin V Apoptosis Detection Kit I, #556547, BD Pharmingen™) was used to measure apoptotic cells by flow cytometry according to the manufacturer’s instructions. Briefly, both floating and adherent cells were harvested, adherent cells were collected by adding a warm solution of 10 mM ethylenediaminetetraacetic acid (EDTA) in PBS, the cells were centrifuged at 500 g for 5 min, washed with ice cold 1× PBS twice and re-suspended in 400 μL 1× binding buffer containing 5 μL Annexin V-FITC and 5 μL PI for 15 min at room temperature in the dark. Fluorescence was induced on a Beckman Coulter Cytomics Quanta SC MPL flow cytometer (10,000 events per sample). Spectral compensation was performed using Cell Lab Quanta analysis software. The number of viable and apoptotic cells were quantified by events in the quadrants. The results were expressed as the percentage of apoptotic cells at the early stage (PI negative and Annexin V positive, lower right quadrant), apoptotic cells at the late stage (PI positive and Annexin V positive, upper right quadrant), necrotic cells (PI positive and Annexin V negative, upper left quadrant) and viable cells (PI negative and Annexin V negative, lower left quadrant).

### 4.12. Cell Uptake

Cellular uptake of cisplatin was quantified by using an ion coupled plasma mass spectrometer (ICP-MS, Agilent Technologies, Tokyo, Japan). MDA-MB-231 cells (50 × 10^4^ cells/flask) were seeded in 25 cm^2^ flasks overnight. Cells were exposed to cisplatin (166 μM) for 24 h under normoxia and hypoxia. On following day, the medium was aspirated, the cells were rinsed with cold PBS, detached using trypsin-EDTA, aliquoted in duplicate in 1.5 mL micro-centrifuge tubes and pelleted by centrifugation at 500 *g* for 5 min. One of each duplicate cell pellet was digested with 20% (*v*/*v*) HNO_3_ overnight at 60 °C and analyzed for Pt(II) content by ICP-MS. The other duplicate was lysed using RIPA lysis buffer that was supplemented with 0.1 mM phenylmethylsulfonyl fluoride (PMSF) (Sigma-Aldrich), a protease Inhibitor Cocktail Set III, Animal-Free—Calbiochem (#535140, Millipore), and a phosphatase Inhibitor Cocktail Set II (#524625, Millipore) and quantified for protein content using the BCA protein assay kit (Pierce, Rockford, IL, USA). The cell uptake is expressed as ng cisplatin/μg cell protein.

### 4.13. Statistical Analysis

The statistical analysis was performed by Graphpad Prism (version 5.00, Graphpad Software Inc., La Jolla, CA, USA). Statistical analysis was performed either using unpaired Student’s *t* test or one-way ANOVA (analysis of variance) with Tukey post-test analysis. Statistical significance is denoted by (*p* < 0.05). All graphs represent the average of at least three independent experiments with triplicates, unless mentioned otherwise in the text, or graphs. Results were represented as mean ± standard deviation (SD).

## 5. Conclusions

Our findings have shown that STAT3 activation, but not HIF-1α, appears to mediate HICR to cisplatin in MDA-MB-231 cells. Additional studies in other TNBC cell lines and primary samples are required to validate the overexpression of STAT3 (rather than HIF-1α) as a biomarker of chemoresistance to cisplatin or as a therapeutic target to improve TNBC chemosensitivity to cisplatin.

## Figures and Tables

**Table 1:** Primer sequences.

Gene	Forward Primers	Reverse Primers
ABCC1	5′-CTCTATCTCTCCCGACATGACC-3′	5′-AGCAGACGATCCACAGCAAAA-3′
ABCC2	5′-CCCTGCTGTTCGATATACCAATC-3′	5′-TCGAGAGAATCCAGAATAGGGAC-3′
ABCC4	5′-AGCTGAGAATGACGCACAGAA-3′	5′-ATATGGGCTGGATTACTTTGGC-3′
ABCC5	5′-AGTCCTGGGTATAGAAGTGTGAG-3′	5′-ATTCCAACGGTCGAGTTCTCC-3′
ABCC6	5′-AAGGAGGTACTAGGTGGGCTT-3	5′-CCAGTAGGACCCTTCGAGC-3′
ABCB1	5′-TTGCTGCTTACATTCAGGTTTCA-3	5′-AGCCTATCTCCTGTCGCATTA-3
GAPDH	5′-GGAGCGAGATCCCTCCAAAAT-3′	5′-GGCTGTTGTCATACTTCTCATGG-3′

## Supplementary Materials

The following are available online at www.mdpi.com/2072-6694/9/10/137/s1. Figure S1: There was no significant difference in clonogenic potential between cells grown in hypoxia or normoxia in the absence of cisplatin. Figure S2: Bar graph illustrating quantification of the Western blot densitometry analysis for Figure 4C. Figure S3: Successful knockdown of HIF-1α is not effective in the reversion of hypoxia-induced cisplatin resistance in the MDA-MB-231 cells. Figure S4: Successful knockdown of HIF-1α is not effective in the reversion of hypoxia-induced cisplatin resistance in the MDA-MB-231 cells regardless of scheduling. Figure S5: Successful knockdown of HIF-1α with siRNA under hypoxia did not enhance cisplatin-induced apoptosis regardless of scheduling in the MDA-MB-231 cells. Figure S6: Stabilization of HIF-1α in normoxia using cobalt chloride as a hypoxia mimetic agent failed to induce cisplatin resistance. Figure S7: qRT-PCR results of ABCC1 and ABCC5 expression in the MDA-MB-231 cells after HIF-1α, and STAT3 knockdown.
