# Supplementary material for: STAT3 but Not HIF-1α Is Important in Mediating Hypoxia-Induced Chemoresistance in MDA-MB-231, a Triple Negative Breast Cancer Cell Line
Source: Cancers (Basel). 2017 Oct 14;9(10):137. doi: 10.3390/cancers9100137 (PMC5664076; doi:10.3390/cancers9100137)
Supplement: Supplementary file 1 [file cancers-09-00137-s001.pdf]

# Supplementary Materials: STAT3 but Not HIF-1α Is Important in Mediating Hypoxia-Induced Chemoresistance in MDA-MB-231, a Triple Negative Breast Cancer Cell Line

**Hoda Soleymani Abyaneh, Nidhi Gupta, Aneta Radziwon-Balicka, Paul Jurasz, John Seubert, Raymond Lai and Afsaneh Lavasanifar**

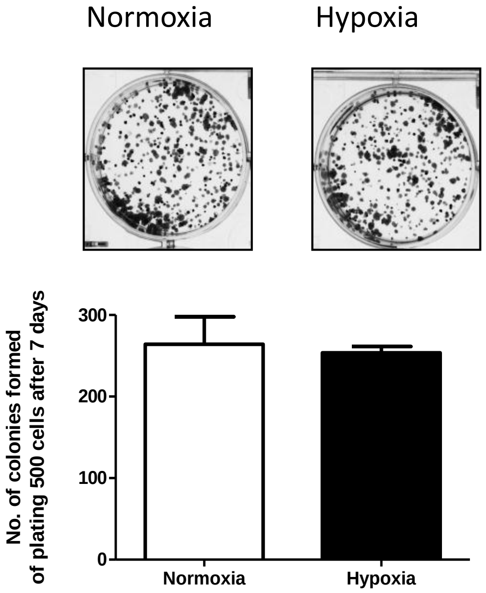


**Figure S1.** There was no significant difference in clonogenic potential between cells grown in hypoxia or normoxia in the absence of cisplatin. Clonogenic survival assay was conducted for MDA-MB-231 cells incubated under hypoxia or normoxia (24 h) in duplicate with plating of 500 cells. The number of colonies formed under normoxia from 500 cells was graphed after 7 days. Data are represented as mean ± SD ($n$=3), (Student's $t$ test, $p$>0.05).

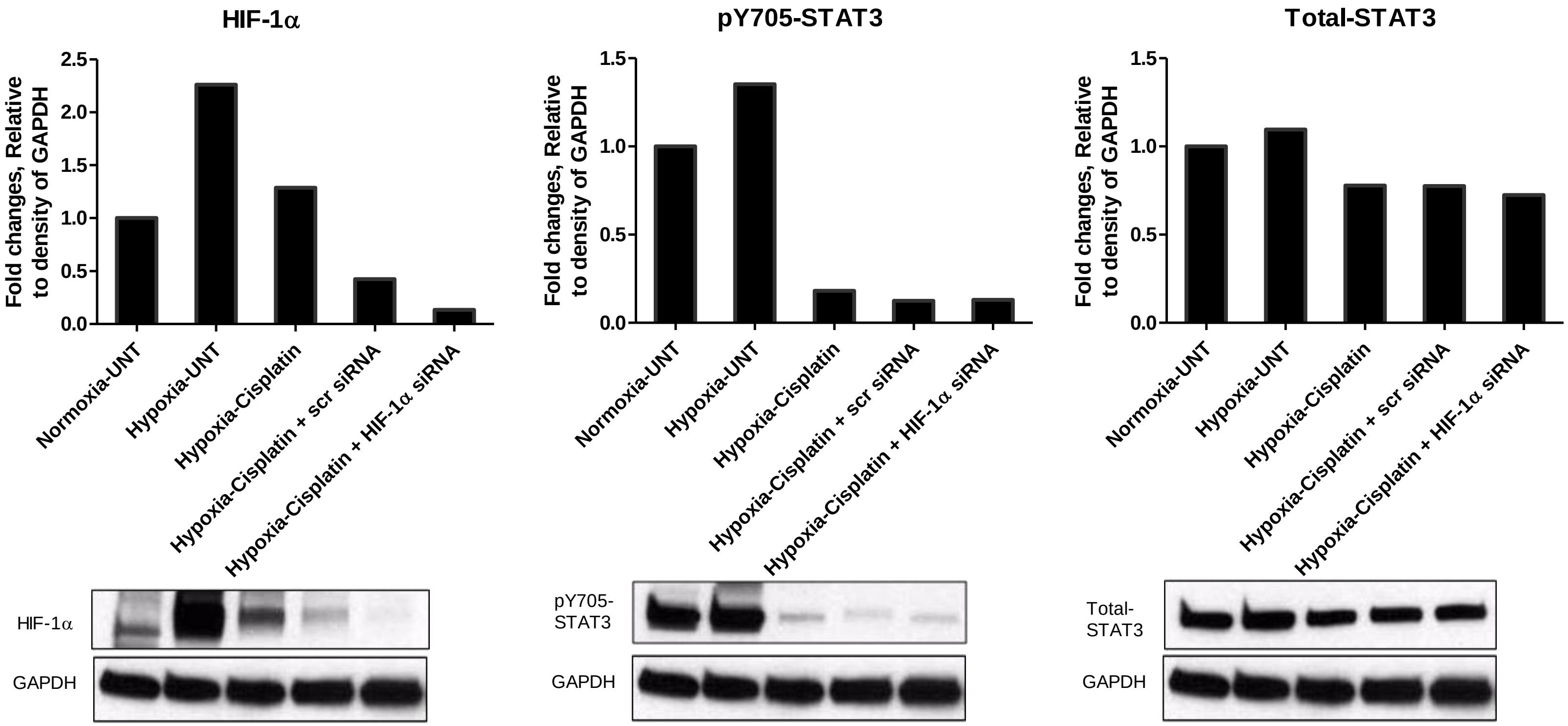


**Figure S2.** Bar graph illustrating quantification of the Western blot densitometry analysis for Figure 4C. In Figure 4C, we intentionally over-exposed the film such that we were able to demonstrate the lack of change in pSTAT3 in lanes 3–5. Densitometry data are expressed as fold changes compared to untreated normoxic group, normalized to GAPDH band intensity.

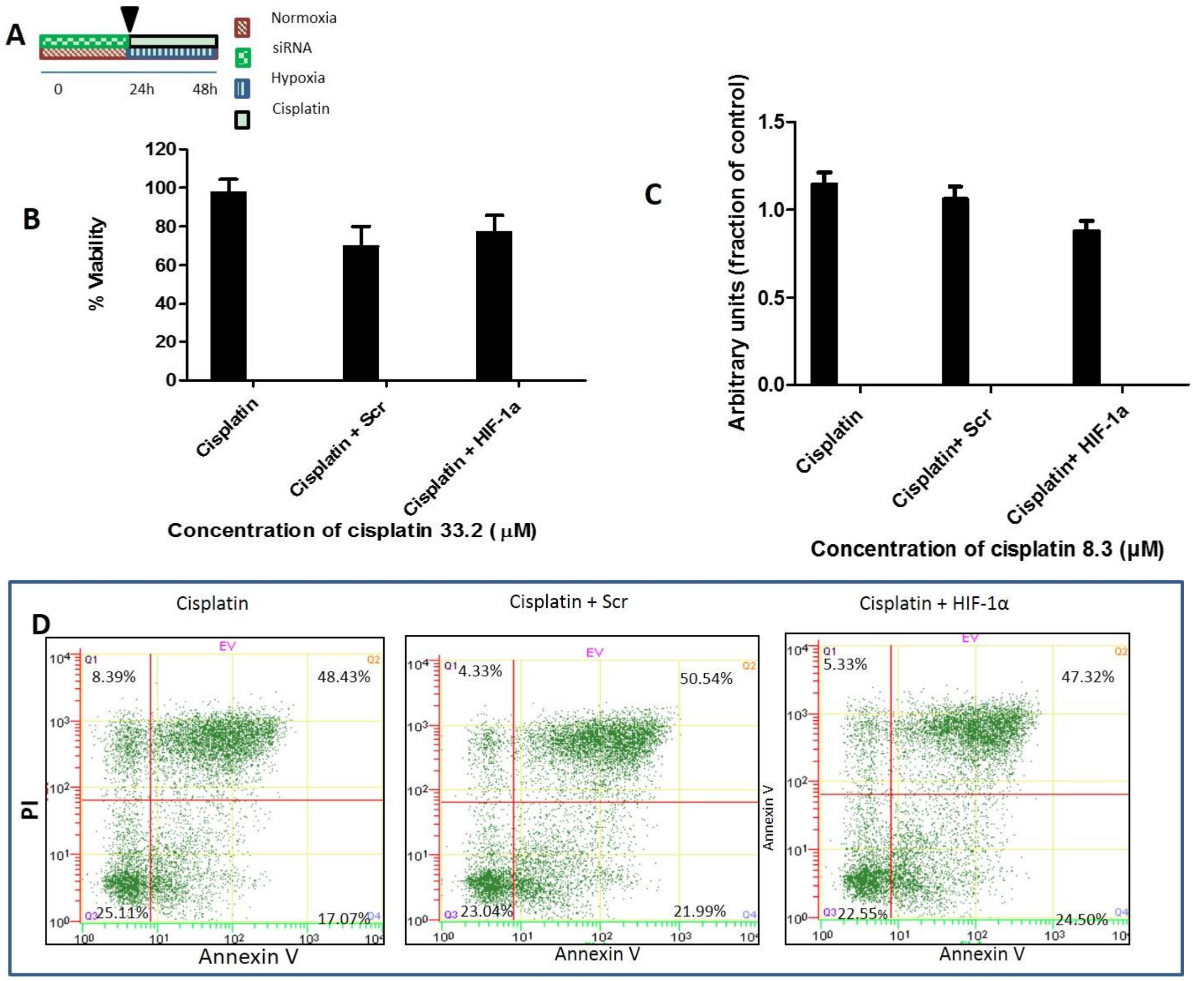


**Figure S3.** Successful knockdown of HIF-1*α* is not effective in reversion of hypoxia-induced cisplatin resistance in the MDA-MB-231 cells. (**a**) Drug treatment diagram for Figures b-d, (**b**) Viability of cells was measured by MTT assay after cells were transfected with HIF-1*α* siRNA under normoxia (24 h) and then treated with cisplatin (33.2 μM) under hypoxia (24 h). (**c**) Clonogenic survival assay was conducted in duplicate with plating 500 cells after cells were transfected with HIF-1*α* siRNA under normoxia (24 h) and then treated with cisplatin (33.2 μM) under hypoxia (24 h). The number of colonies formed (% of control) from 500 cells was graphed after 10 days. Data are represented as mean ± SD ($n$=3). No significance difference was observed between the groups (one way ANOVA followed by a *post-hoc* Tukey test, $p$>0.05). (**d**) Apoptotic behaviour of cells was measured by Annexin V/PI assay for cells transfected with HIF-1*α* siRNA under normoxia (24 h) and then treated with cisplatin (33.2 μM) under hypoxia (24 h). Flow cytometry analysis showed different populations of (Q1) necrotic or already dead cells (PI positive), (Q2) cells in end stage apoptosis (FITC Annexin V and PI positive), (Q3) viable cells (FITC Annexin V and PI negative), and (Q4) cells in early stage of apoptosis (FITC Annexin V positive and PI negative). No significance difference was observed between the groups treated with HIF-1*α* and scrambled siRNAs for different populations of the cells (Q1–Q4), (Student's $t$ test, $p$>0.05) ($n$=3).

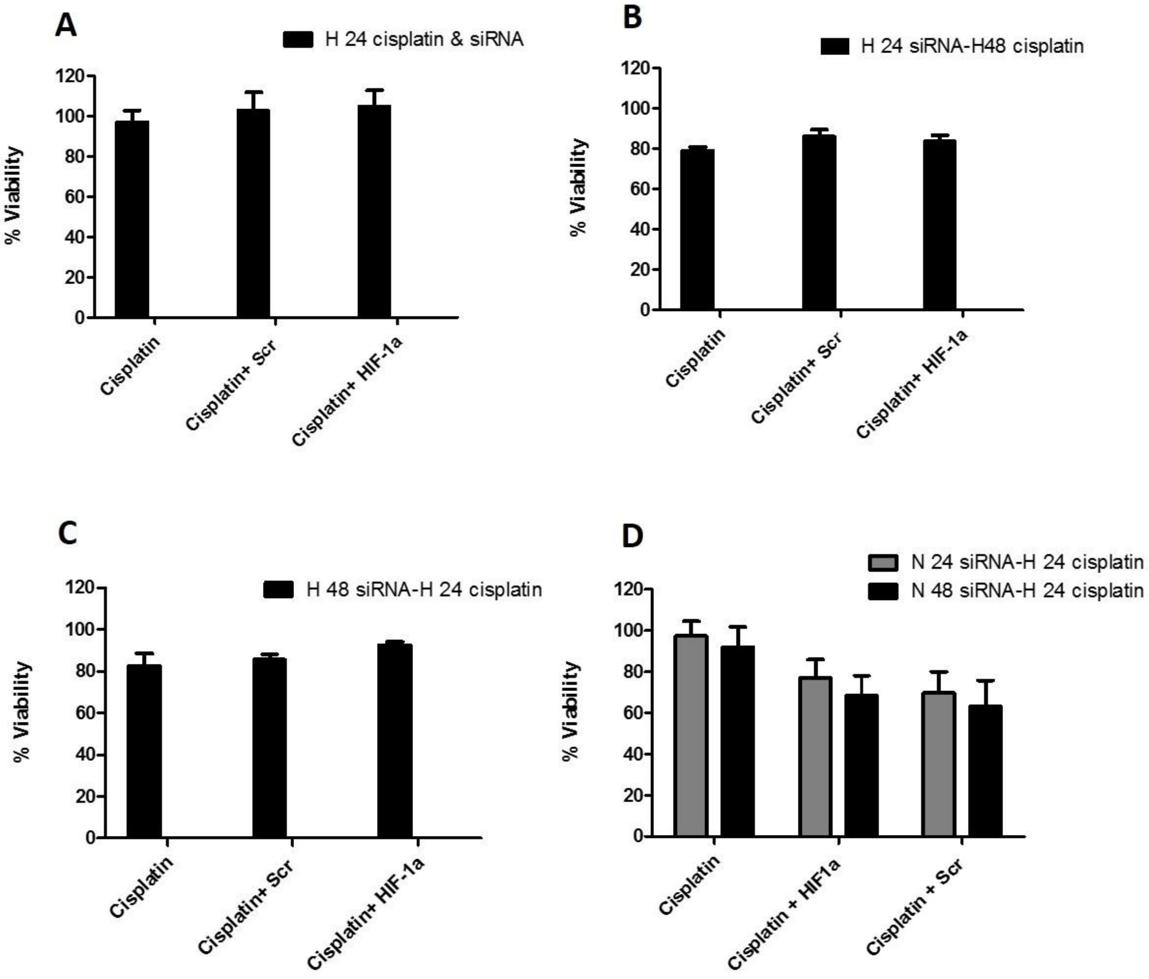


**Figure S4.** Successful knockdown of HIF-1$\alpha$ is not effective in reversion of hypoxia-induced cisplatin resistance in the MDA-MB-231 cells regardless of scheduling. Viability of cells was measured by MTT assay after (**a**) cells were transfected with HIF-1$\alpha$ siRNA and concurrently treated with cisplatin (33.2 μM) for 24 h under hypoxia (H), (**b**) cells were transfected with HIF-1$\alpha$ siRNA under hypoxia (24 h) and then treated with cisplatin (33.2 μM) for 48 h under hypoxia, (**c**) cells were transfected with HIF-1$\alpha$ siRNA under hypoxia (48 h) and then treated with cisplatin (33.2 μM) for 24 h under hypoxia, and (**d**) cells were transfected with HIF-1$\alpha$ siRNA under normoxia (N) for 24 h and then treated with cisplatin (66.4 μM) for 24 or 48 h under hypoxia. No significance difference was observed between the groups (one way ANOVA followed by a *post-hoc* Tukey test, $p$>0.05) ($n$=3).

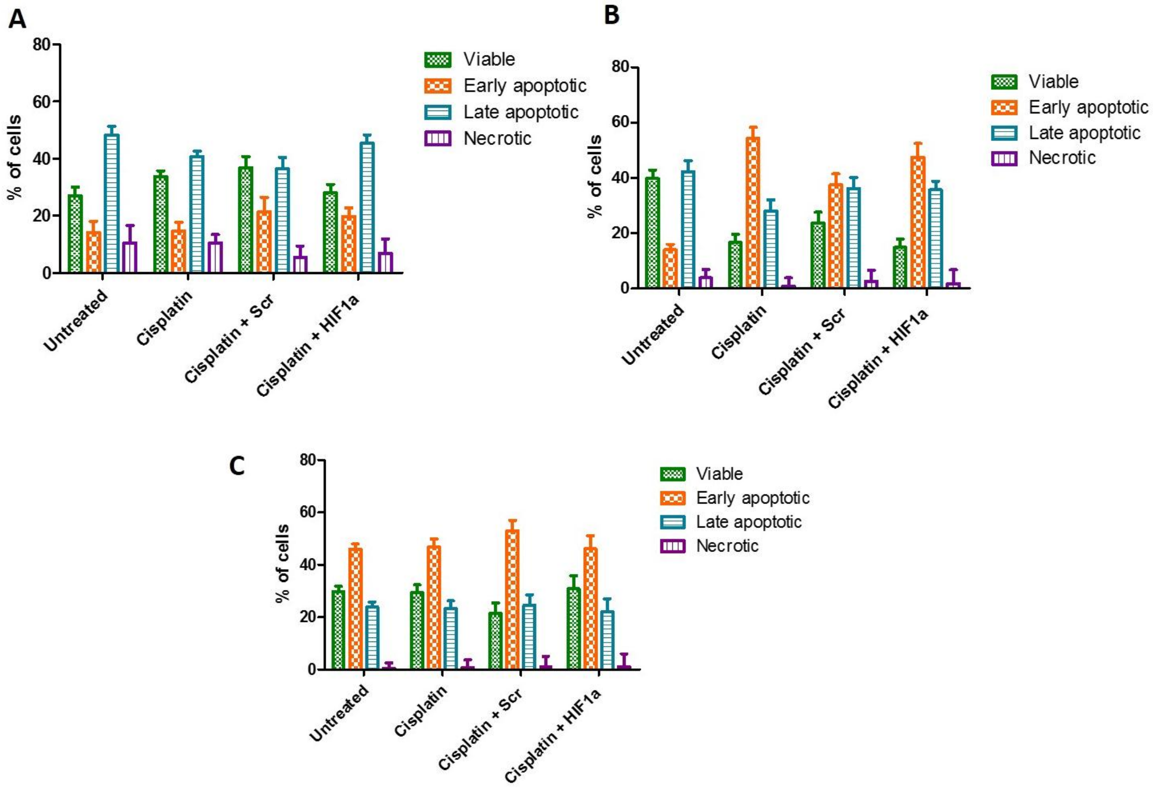


**Figure S5.** Successful knockdown of HIF-1$\alpha$ with siRNA under hypoxia didn't enhance cisplatin-induced apoptosis regardless of scheduling in the MDA-MB-231 cells. Apoptotic behaviour of cells was measured by Annexin V/PI assay for cells (**a**) concurrently transfected with HIF-1$\alpha$ siRNA and treated with cisplatin (33.2 μM) for 48 h under hypoxia, (**b**) transfected with HIF-1$\alpha$ siRNA under hypoxia (24 h) and then treated with cisplatin (33.2 μM) for 48 h under hypoxia, (**c**) transfected with HIF-1$\alpha$ siRNA under hypoxia (48 h) and then treated with cisplatin (33.2 μM) for 24 h under hypoxia. Flow cytometry analysis showed different populations of necrotic or already dead cells (PI positive), cells in end stage apoptosis (FITC Annexin V and PI positive), viable cells (FITC Annexin V and PI negative), and cells in early stage of apoptosis (FITC Annexin V positive and PI negative). No significance difference was observed in the percentage of different populations (viable, early apoptotic, late apoptotic and necrotic) between cells transfected with HIF-1$\alpha$ and scrambled siRNAs, (Student's *t* test, $p>0.05$) ($n=3$).

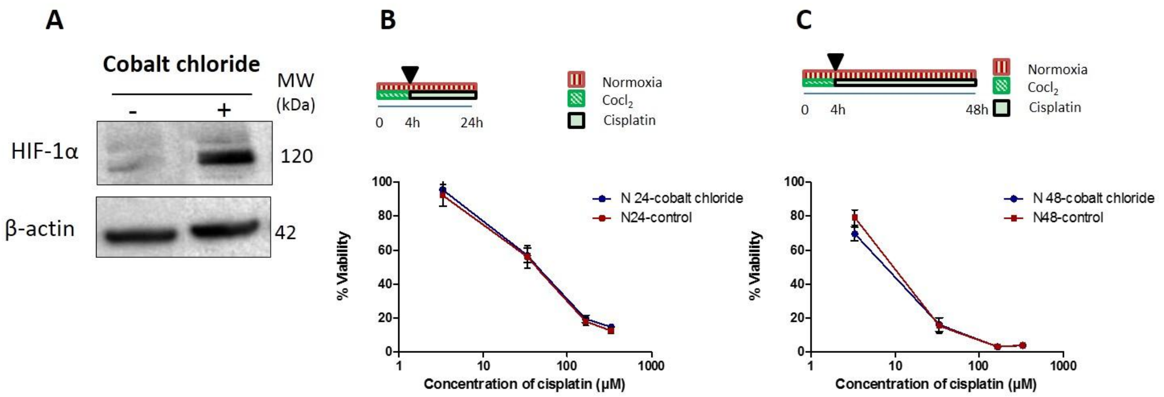


**Figure S6.** Stabilization of HIF-1$\alpha$ in normoxia using cobalt chloride as hypoxia mimetic agent failed to induce cisplatin resistance. (**a**) Expression of HIF-1$\alpha$ was measured by immunoblotting after MDA-MB-231 cells were treated with $CoCl_2$ (100 μM) for 4 h under normoxia. (**b-c**) Viability of cells was measured by MTT assay for cells treated with cisplatin after using cobalt chloride as hypoxic

mimicking agent. Cells first treated with cobalt chloride (100 μM) for 4 h. Then media was replaced and cells were exposed to increasing concentration of cisplatin for (**b**) 24 and (**c**) 48 h under normoxia (N). No significance difference was observed between groups (*n*=3).

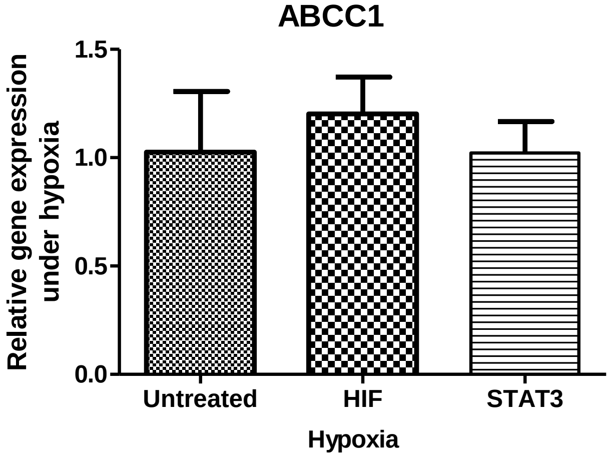


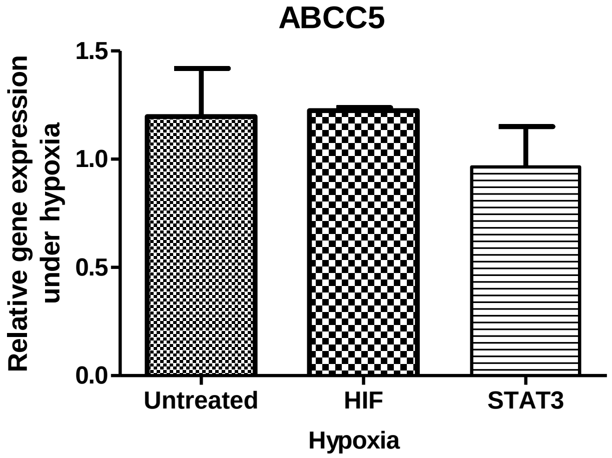


**Figure S7.** qRT-PCR results of ABCC1 and ABCC5 expression in the MDA-MB 231 cells after HIF-1$\alpha$, and STAT3 knockdown. Cells were treated with scrambled (Scr), HIF-1$\alpha$, and STAT3 siRNAs under normoxia for 24 h, and then incubated under hypoxia for 48 h. The RT-PCR results were normalized to GAPDH, and further normalized to untreated hypoxic sample. No significance difference was observed as compared to untreated hypoxic group (Student's t test, $p$>0.05) ($n$=3).
